# A COMPARATIVE STUDY OF FALL RISK ASSESSMENT IN COMMUNITY DWELLING OLDER ADULTS IN INDIA

**DOI:** 10.1093/geroni/igac059.3093

**Published:** 2022-12-20

**Authors:** Snehal Kulkarni

**Affiliations:** Savitribai Phule Pune University, Pune, Maharashtra, India

## Abstract

Falls in older adults can be prevented with early identification. Recent research investigates the use of wearable sensors to provide quantitative data on mobility parameters to assess risk of fall. However, the efficiency of these new methods in comparison to conventional fall risk assessment tools is unknown. Therefore, the current study compares the sensor-based fall risk assessment with conventional fall risk measures. The study included 659 community-dwelling older adults (>60 years) who were followed for 12 months after baseline. The American Geriatric Society Fall Risk Assessment tool was used to assess risk of fall conventionally and Kinesis QTUG was used to assess the fall risk using sensors. Receiver operating characteristics curve (ROC) was used to compare the sensor-based method and the conventional method of assessing fall risk. Out of the 659 community-dwelling older adults, 24% (163) of older adults reported a fall in 12 months. According to the sensor-based assessment, 23% of older adults had low risk, 50% had medium risk and 27% had high risk of fall and according to the conventional method 66.1% were in low risk, 27.1% were in medium risk and 6.8% were in high risk. The ROC analysis showed that the sensor-based methods (AUC-78%; sensitivity- 93.8% specificity – 56.3%) outperformed the conventional method (AUC – 61.2%; sensitivity – 82.8% and specificity - 67.2%) of identifying older adults at risk of fall. Therefore, use of simple wearable sensors can determine the risk of future falls.

